# Environmentally friendly power generator based on moving liquid dielectric and double layer effect

**DOI:** 10.1038/srep26708

**Published:** 2016-06-03

**Authors:** D. H. Huynh, T. C. Nguyen, P. D. Nguyen, C. D. Abeyrathne, Md. S. Hossain, R. Evans, E. Skafidas

**Affiliations:** 1 Centre for Neural Engineering, Bld 261, 203 Bouverie St, The University of Melbourne, Parkville, VIC 3010, Australia; 2 Department of Electrical and Electronic Engineering, Bld 193, Wilson Avenue, The University of Melbourne, Parkville, VIC 3010, Australia; 3 NICTA Victoria Research Laboratory, 115 Batman Street, West Melbourne VIC 3003, Australia

## Abstract

An electrostatic power generator converts mechanical energy to electrical energy by utilising the principle of variable capacitance. This change in capacitance is usually achieved by varying the gap or overlap between two parallel metallic plates. This paper proposes a novel electrostatic micro power generator where the change in capacitance is achieved by the movement of an aqueous solution of *NaCl*. A significant change in capacitance is achieved due to the higher than air dielectric constant of water and the Helmholtz double layer capacitor formed by ion separation at the electrode interfaces. The proposed device has significant advantages over traditional electrostatic devices which include low bias voltage and low mechanical frequency of operation. This is critical if the proposed device is to have utility in harvesting power from the environment. A figure of merit exceeding 10000(10^8^*μW*)/(*mm*^2^*HzV*^2^) which is two orders of magnitude greater than previous devices, is demonstrated for a prototype operating at a bias voltage of 1.2 *V* and a droplet frequency of 6 *Hz*. Concepts are presented for large scale power harvesting.

There is a significant research effort focused on producing new higher efficiency and power density mechanical energy harvesting systems. Many small scale prototypes have been demonstrated and there is great potential that these concepts may in the future be realised for large-scale energy harvesting. To date, the three most widely investigated mechanical energy harvesting methods are based on: piezoelectric^1^,^2^,^3^,^4^,^5^, triboelectric^6^,^7^,^8^, and electrostatic^9^,^10^,^11^,^12^ principles. Piezoelectric and triboelectric generators are the most popular, with piezoelectric generators achieving power densities of 160 *Wm*^−2 ^2 whilst triboelectric generators with power densities of 500 *Wm*^−2^^13^ have been demonstrated.

Notwithstanding the exciting progress there are some major drawbacks that have hindered the progress of electrostatic generators. These include the requirement for high bias voltages, some as high as many hundreds of volts^14^,^15^,^16^, complex electrical circuits and high voltage device insulation required for safety in practical applications. The efficiency of devices can also be limited as they can usually only harvest vibrational or kinetic energy along a single axes of motion. The vibration frequencies required to achieve acceptable power harvesting are very high, usually in the order of a hundred Hz to kHz^17^,^18^,^19^ which makes them less than ideal for environmental power harvesting. In order to overcome these limitations a new and highly innovative electrostatic harvesting liquid electrode device was proposed in T. Krupenkin and J. A. Taylor^20^. Unfortunately the proposed liquid metal electrode was constructed from mercury which is a highly toxic with extensive evidence outlining mercury’s deleterious effects on human health and the environment.

Here we proposed a novel electrostatic generator that utilises a moveable liquid electrolytic dielectric. Although the use of a liquid dielectrics for energy generation have been recently reported^21^,^22^,^23^,^24^, here we propose a new ionic liquid dielectric device that not only takes advantage of the difference of dielectric constant of water versus air but also uses the free ions to generate a significantly larger capacitance change due to the formation of a Helmholtz double layer due to the bias voltage applied at the electrodes.

The ionic liquid electrolytic dielectric offers many desirable attributes including a flexible conformal material that is biocompatible, is abundant (sea water) and most importantly has a larger dielectric constant, much higher than air, resulting in higher energy harvesting capability. We further improve device performance by facilitating the sliding motion of the ionic fluid over the electrode surface by increasing surface hydrophobicity by depositing a thin layer *TiO*_2_ over the electrode surface. The proposed device has significant advantages over traditional electrostatic devices in term of cost, simplicity of fabrication, does not require any specific high cost packaging or vacuum shielding, and is environmentally friendly. Importantly, the prototype device possess a very high surface charge density per conversion cycle of 3.8 *mC*/*m*^2^ and achieves a figure of merit exceeding10000(10^8^*μW*)/(*mm*^2^*HzV*^2^), which is two orders of magnitude greater than previously reported devices.

This paper reports on the design, fabrication and characterisation of an ionic liquid dielectric power harvesting generator device. Potential applications of this device are described, including harvesting energy from droplets (raindrops), hydropower or water waves.

## Principle of operation

**Figure 1:**
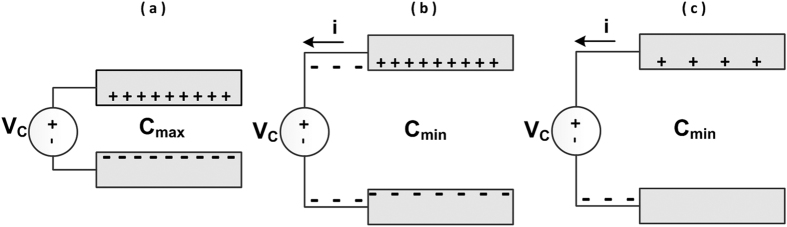
Principle operation of a typical gap changing variable capacitor: (**a**) represents the situation when the gap is minimal and capacitance is maximal, (**b,c**) describe the situation when the gap is maximal and capacitance is reduced to the minimal value, electrons are transferred to maintain constant voltage and hence current is generated.

Traditionally, electrostatic micro generator devices achieve changing capacitance by varying the gap or overlap area between two parallel plates of the capacitor. As shown in Fig. 1(a), the capacitance is maximized when the gap is smallest. In Fig. 1(b), when an external mechanical force is applied, the gap between the plates is increased resulting in reduced capacitance. If the voltage applied to the two terminals of the variable capacitor is fixed (by external sources such as a battery, a pre-charged capacitor or other active circuitry), the change in capacitance during this transition will generate an electric current as indicated in Fig. 1(c). The total amount of energy produced over a cycle is equal to^25^:


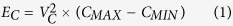


**Figure 2:**
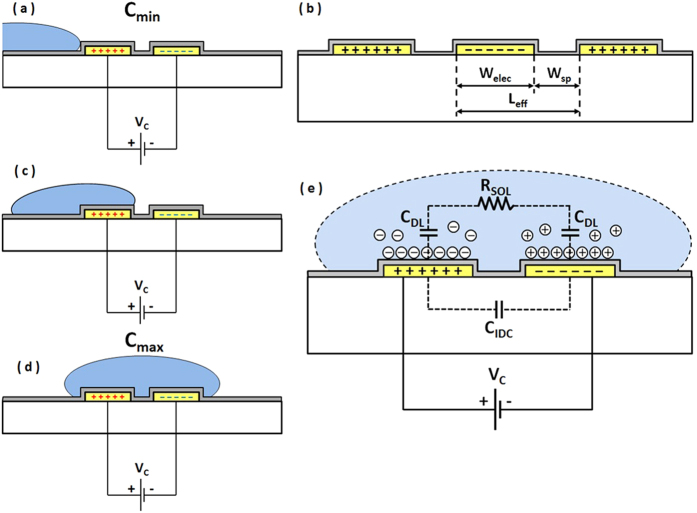
Principle operation of proposed device (yellow layer represents interdigitated metal fingers and grey layer represents oxide layer, (**a,c,d**) represents droplet (blue color) movement on hydrophobic surface during transition state, (**b**) indicates factors which affect geometric capacitance, (**e**) shows the double layer effect and its equivalent electrical model.

To increase the energy produced per cycle, a higher bias voltage or larger change in variable capacitance is required. In practice, it is difficult to achieve significant change in capacitance due to limited change in displacement of the solid plate electrodes. Therefore to increase generated power, higher bias voltages (*V*_*C*_), up to 1000 *V*^14^, are employed. This introduces many challenges. Our proposed device, while still based on the principle of variable capacitance, utilises the displacement of a liquid dielectric rather than solid electrodes. Figure 2(b) shows the main factors affecting geometric capacitance of the device; these include electrode length, width, spacing between electrodes, dielectric constant of substrate and medium above the electrodes. Analytical expression relating these geometric parameters to capacitance can be found in Van Gerwen, P. *et al*.^26^.

The addition of a hydrophobic oxide surface over the electrode facilitates droplet motion. As can be seen from Fig. 2(a,c), when the droplet is not over the surface of the electrodes, the capacitance is only due the interdigitated capacitance. This corresponds to the minimum capacitance value *C*_*MIN*_. Figure 2(d) illustrates the situation when droplet is fully covering the electrode area. At this stage, the device capacitance is at its maximum value *C*_*MAX*_. This change in capacitance is due to two factors. Firstly, the dielectric constant of the medium on top of the electrodes has increased significantly. For pure water at low frequency, its dielectric constant is approximately 78 times higher than that of the air^27^. Secondly, water droplets with dissolved salt (*NaCl*) contain charged ions. These ions accumulate at the interface of the metal electrodes and liquid electrolyte when a potential difference is applied. These layers of accumulated charge create an electrical double layer, which results in a significant, orders of magnitude, change in capacitance. Under a potential difference between two electrodes, positive ions are accumulated at the surface of negative electrode and negative ions are accumulated at the surface of positive electrode simultaneously. This capacitance due to charge separation can be modelled as a parallel plate capacitor with very small gap. An estimate of this capacitance can be obtained by^28^:


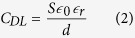


where *S* is the area of electrodes that interface with the solution and *d* is the effective thickness of the double layer. This dielectric layer thickness is referred as the Debye length. The major factors that affect the Debye length are ion concentration, ion charge and temperature. For a specific ion at a certain temperature, a higher ion concentration will result in a smaller Debye length. Figure 2(e) shows the schematic concept of this double layer effect and its equivalent electrical model. In this model, *R*_*SOL*_ represents the series equivalent resistance of the double layer. This equivalent resistor also depends on ion concentration of the electrolyte, device geometry, and temperature^28^.

### Device fabrication and measurement setup

#### Device fabrication

The fabrication process of the liquid electrolyte generator involved the following steps:

Glass slides (Sail Brand) were used as the substrate layer and a quartz wafer was used to produce a mask. A thin film (100 *nm*) of Chromium (Cr) was deposited on the glass slides and on the quartz wafer by Electron beam evaporation (Inte vac Nanochrome II model). The lithographic features on the Cr coated quartz mask were produced using laser ablation (SUSS SLP300 model).

**Figure 3:**
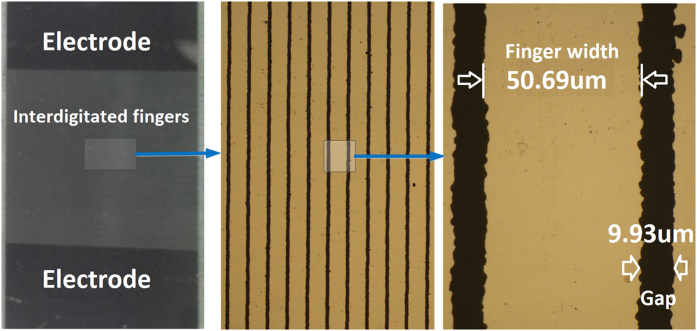
Image of a device after fabrication, (**a**) shows the device at large scale, (**b,c**) represent the interdigited finger with dimensions.

To pattern the interdigitated capacitor with finger width of 50 *μm* and finger gap of 10 *μm*, *AZ*1512HS resist was deposited on the slides, spun at 3000 *rpm* for 1 minute, resulting in a resist thickness of approximately 1.5 *μm*. The resist was exposed using the Cr coated quartz masks to UV light of 356 *nm* wavelength and power of 30 *mWcm*^−2^ for an exposure time of 15 seconds. After exposure, the sample was developed for 90 second using *AZ*712: *H*_2_*O* (3:2) developer and then rinsed with deionized water and dried using a nitrogen stream. The slide was then wet etched using a Cr etchant to define the metal interdigitated structure. The sample was then cleaned with acetone in an ultrasonic bath, rinsed with IPA and then dried with a nitrogen gun. In order to create the isolation between the metal fingers and the liquid dielectric, a layer of oxide was deposited. Kapton tape was used to cover the metal pads. A 10 *nm* layer of titanium dioxide (*TiO*_2_) was deposited using atomic layer deposition (ALD) method (Fiji F200 from Cambridge Nanotech). TiO_2_ was chosen instead of conventional *SiO*_2_ due to its inherit hydrophobicity^29^. An image of the fabricated device is shown in Fig. 3.

#### Measurement setup

**Figure 4:**
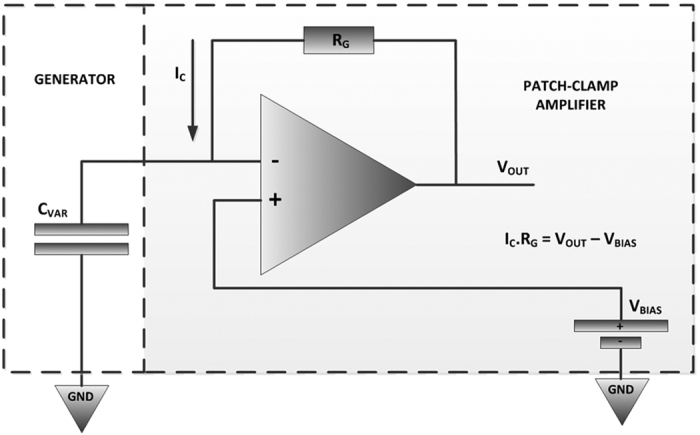
Constant voltage measurement setup: *V*_*BIAS*_ set a constant voltage between 2 terminals of the variable capacitor *C*_*VAR*_ generated current *I*_*C*_ is converted to output voltage *V*_*OUT*_ by resistor *R*_*G*_.

Figure 4 shows the schematic of a constant voltage measurement setup for our proposed devices. Voltage across two terminals of the variable capacitor *C*_*VAR*_ was fixed at *V*_*BIAS*_ by using a voltage-clamp amplifier circuit. The charge stored on this variable capacitor is equal to:


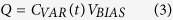


Varying capacitance whilst keeping *V*_*BIAS*_ fixed results in the generation of current *I*_*C*_. This current is measured using a trans-impedance amplifier which converts it to voltage using a resistor *R*_*G*_.

## Results and Discussion

### Constant voltage measurement using a patch clamp amplifier

**Figure 5:**
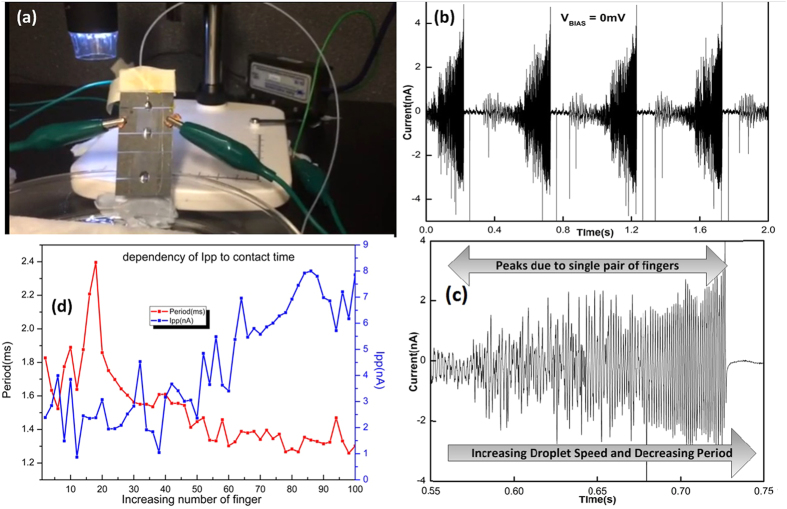
Measurement setup for sliding water droplet. (**a**) shows a typical test setup, (**b**) indicates current generated when drop pass through device at 0 *mV* bias voltage, (**c**) represent a closer look at current generated, (**d**) shows the dependency of peak current on contact time.

Figure 5(a) illustrates a test setup used to verify the functionality of the device. The device was fixed at an angle of approximately 60°. A Kd Scientific micro pump was used to generate small droplets of 18 *M*Ω water. The patch clamp amplifier (Model MultiClamp 700B) was used to clamp the voltage and to monitor the generated current. As can be observed from Fig. 5(b), current is generated when the droplet slides over the device and there is negligible current generated after droplet had passed over the effective area of the device. Figure 5(c) represents a magnified section of the output current which illustrates the current generated when the droplet was passing through each of the 50 *μm* pair of finger. The magnitude of peak current is increased as the droplet’s speed is increased. As droplets travel across the fingers the peak current generated is plotted in Fig. 5(d). This graph indicates a strong inversely proportional relationship between the droplet sliding period and the amplitude of generated current. It is consistent with the equation that defines the generated current due capacitance change, which is equal to:


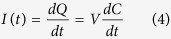


**Figure 6:**
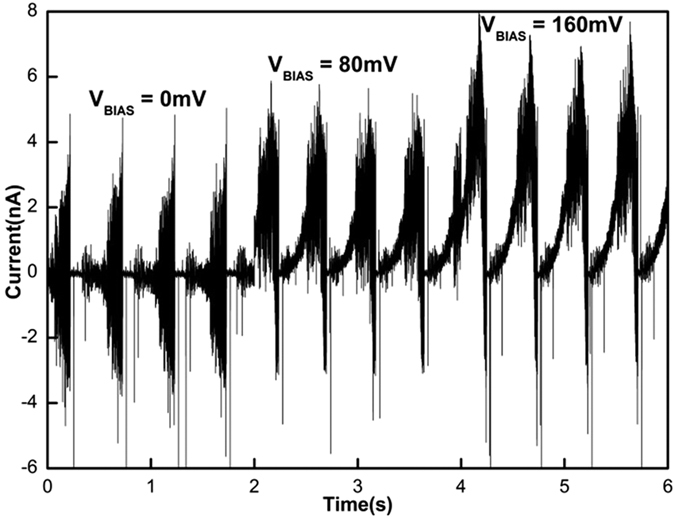
Dependency of output current on bias voltage. Peak current is lowest at 0 *mv* bias voltage and it is increased at 80 *mV* and 160 *mV* bias voltage.

The effect of bias voltage on device performance was also investigated by varying the bias voltage whilst other test conditions, such as size of droplet and the speed of drop, were kept constant. The measurements were performed for three different bias voltages (0 *mV*, 80 *mV* and 160 *mV*). The measurements are shown in Fig. 6. As the bias voltage was increased, there was an increase in peak current generated confirming that the magnitude of current generated depends on the bias voltage.

### A potential application of the device to large scale environmentally friendly energy harvesting

**Figure 7:**
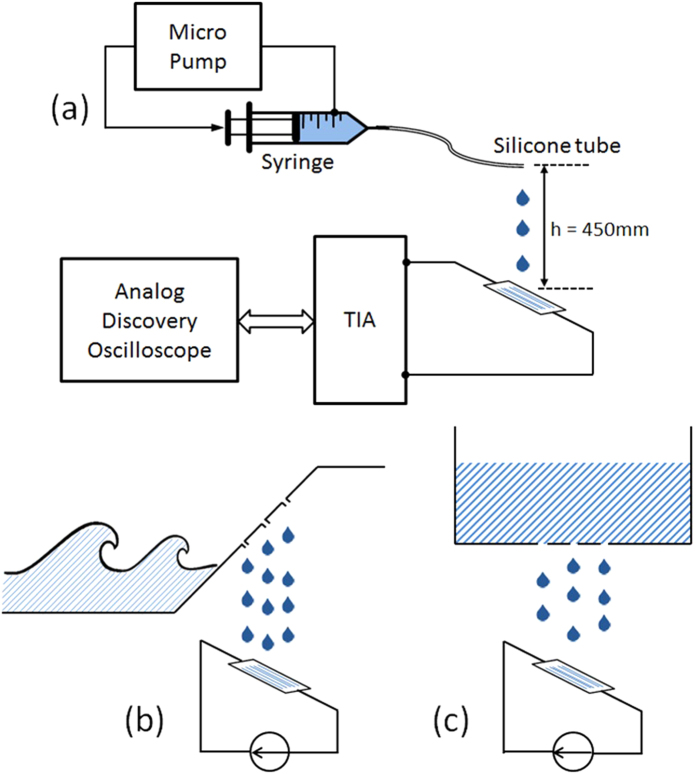
(**a**) Measurement setup for a potential application of the device and concepts of generating droplets from water wave (**b**) and from a water reservoir (**c**).

A promising application of this proposed device is for harvesting energy from liquid droplets. Liquid droplets are abundant in our environment. They can be freely available such as rain drops or can be easily formed in a marine environment by exploiting water wave or from a prefilled water reservoir. The concept of how water droplets are formed for practical applications is outlined in Fig. 7(b,c).

Figure 7(a) indicates details of an experimental setup for harvesting energy from the droplets. The energy harvesting device was placed at a distance of 450 *mm* below the drop generating tube. The bias voltage was set to be at 102 *V*. The gain-setting resistor was selected to be 1.8 *M*Ω. A micro pump was used to generate droplets. The flow rate was 6 *mL* per minute with an average of 6 droplets per second resulting in a drop volume of 16.7 *μL*. The droplet is on the surface of the device with an effective surface area of approximately 7 *mm*^2^.

**Figure 8:**
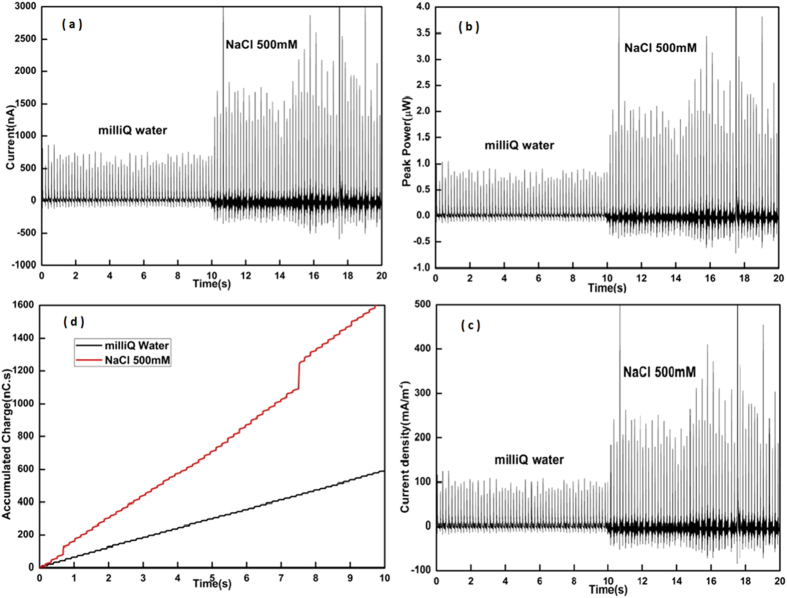
(**a**) Peak current (in *nA*) generated from two different types of droplets (milliQ water and NaCl), discontinuity at 0.5 s and 8 s on NaCl charge curve is due to some abnormal spike of current generated (**b**) Peak power (in *μW* ) associated with current generated (**c**) Current density (in *mA*/*m*^2^) (**d**) Accumulated charge generated over 10 s (in *nC*).

Figure 8 summaries the experimental results. The generated current is shown in Fig. 8(a). Current generated from milliQ water droplets, which have very low ion concentration, and droplets with 500 *mM* NaCl, which have similar concentration to seawater, are overlaid on the same graph for comparison. Current generated varies significantly depending on ion concentration. The peak current observed was approximately 2 *μA* for the 500 *mM* NaCl droplets. Figure 8(d) shows the corresponding current density per surface area of the device. The peak current density of approximately 250 *mA*/*m*^2^ is achieved for a 17 *μL* droplet of 500 *mM* NaCl. Comparison is shown in Table 1.

To estimate average energy and power generated, the charge generated over 10 s was integrated. This result is shown in Fig. 8(c). The average charge generated for milliQ and NaCl solution (500 *mM*) were 55 *nC* and 160 *nC* per second respectively. For droplets with 500 *mM* NaCl the equivalent surface charge density per conversion cycle is (160 *nC*)/(6 × 7 *mm*^2^) = 3.8 *mC*/*m*^2^. And the peak power density is 357 *mWm*^−2^ when operated at a bias voltage of 1.2 *V*. Although the output power (Fig. 8(b)) is lower compared to other electro-static generator devices it is important to emphasize that a much lower operating voltage is used here.

To evaluate and to compare device performance the figure of merit (FOM) proposed by Basset, P. *et al*.^17^ is used. This FOM is defined as:


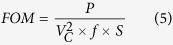


where *P* is the converted power (in *μW*), *V*_*C*_ is maximum voltage applied to variable capacitor (in *V*), *f* is operating frequency (in *Hz*) and *S* is device area (in *mm*^2^). The results, shown in Table 2, indicate that our proposed electrostatic generator has a two order of improvement over previous devices.

### Future improvements

Our device possesses very high surface charge density per conversion cycle and there is a great potential for boosting the output power. Here we discuss on some key factors that affect the amount energy produced and how output power can be improved.

### Electrolyte concentration

Equation (2) describes the principal factors that govern the amount of energy produced by a constant voltage electrostatic generator. At a certain bias voltage *V*_*BIAS*_, the produced energy is directly proportional to the capacitance change. From the model shown in Fig. 4(e), this capacitance change depends on geometric capacitance *C*_*IDC*_, double layer capacitance *C*_*DL*_ and its equivalent series resistance *R*_*SOL*_. While electrolyte concentration has minimal effect on the geometric capacitance, it substantially affects *C*_*DL*_ and *R*_*SOL*_. Hence a higher concentration electrolyte will create a larger overall capacitance change due to a decrease in *R*_*SOL*_ and an increase in *C*_*DL*_.

### Bias voltage

From equation(2), the amount of energy produced is directly proportional to 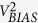. Hence an increase in *V*_*BIAS*_ will significantly improve output power of the proposed device. If the bias voltage is increased to 24 *V*, which is 20 times higher than our test condition, the output power could increase by 400 times. However, a higher bias voltage requires a thicker insulation layer which will reduce the change in capacitance. To increase the bias voltage of the device while still maintaining the total capacitance change, a low dielectric constant and higher breakdown voltage material can be used.

### Structural dimension and surface area of the device

Reducing electrode and gap dimensions and increasing the total surface area of the devices will also significantly increase the capacitance change, which leads to an increase in the produced energy and the output power. Currently, the prototype device has finger width of 50 *μm* and electrode gap of 10 *μm*. This dimension can be reduced to much smaller without increasing the complexity or the cost of fabrication process. Shim *et al*.^30^ recently has reported a cheap and reliable technique to produce large area interdigitated capacitor with gap between fingers is less than 500 *nm* using conventional lithography process. If the device dimension can be reduced to 2.5 *μm* finger width and 0.5 *μm* gap, the capacitance due to its geometry could be improved by 20 times. In addition, the fabrication process can be slightly modified to improve total surface areas of the device. Prior to deposition of metal on substrate, nano-imprinting can be used to increase nanoscale surfaces roughness. With nano-imprint technology, nano structuring with resolution smaller than 10 *nm* has been reported^31^. Assuming nano-imprint mould with pattern of 50 *nm* square and 100 *nm* height is deployed, for every 1 *μm*^2^ area, there will be 100 nano pattern and therefore the total new surface area will be (1 + 100 × 0.05 × 0.10 × 4 × 100 = 3 *μm*^2^), which is equivalent to three times increasing in total surface area. Both geometric capacitance (*C*_*IDC*_) and double layer capacitance (*C*_*DL*_) in equivalent model of Fig. 5(e) are dependent on the surface area of the device. Therefore, the total equivalent capacitance of the device will be significantly increased because of an escalation of the total surface area.

### Contact time

By using nano-imprint to predefine surface roughness, the droplet contact angle will also be increased and the droplet sliding angle will be decreased^32^,^33^. This will lead to shorter contact time when droplet is on the device surface and hence the generated current will be increased.

### Optimal load value

**Figure 9:**
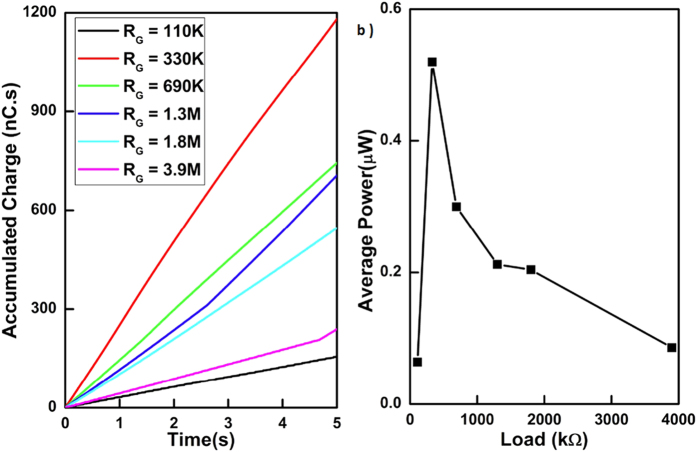
Load dependency of the device (**a**) Accumulated charge over 5 seconds per different load (**b**) Average power generated for milliQ water droplet at different load condition with *V*_*C*_ = 2 *V.*

Further experimentation was carried out to investigate the effect of load on the power generated. Matching between the load and the device impedance will produce maximum output power. In this experiment, the load resistor was varied from 110 *k*Ω to 3.9 *M*Ω. MilliQ water(18 *M*Ω) droplets were used for this experiment, with a drip rate of approximately 7 droplets per second and bias voltage, applied on variable capacitor, was set at 2 *V*. Figure 9 summaries the test results. Figure 9(a) represents the total amount of charge generated over period of 5 second. Figure 9(b) shows the estimated average power generated from this generated charge. This result highlights that the output power of the device is load dependent. The output power for varying loads can differ by a factor of 5 times. Therefore, by carefully optimising the load condition, the output power of this generator can be substantially improved.

## Conclusions

We have proposed a novel electrostatic power generator that converts mechanical energy to electrical energy by utilising movable ionic liquid electrolyte as the dielectric. This device offers many advantages over the traditional electrostatic generators. It exhibits large capacitance change due to the electrical double layer effect formed by ion separation and voltages applied at the electrodes and high dielectric constant of water. The achieved surface charge density and current density are favorable when compared to previous reported electrostatic generators. The fabrication process of this device only involves conventional lithography step which is cheap, scalable to macro size and does not require any high cost packaging or vacuum shielding. A peak current density of 250(*mA*)/(*m*^2^) and FOM of 
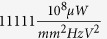
 have been demonstrated for a prototype with the electrode width and gap of 50 *μm* and 10 *μm* respectively operating at 1.2 *V* and a droplet dropping rate of 6 *Hz*. The paper demonstrates a novel concept of an electrostatic generator device that can be used to harvest many widely available sources of (ionic) saline water droplets.

## Additional Information

**How to cite this article**: Huynh, D. H. *et al*. Environmentally friendly power generator based on moving liquid dielectric and double layer effect. *Sci. Rep.* **6**, 26708; doi: 10.1038/srep26708 (2016).

## Figures and Tables

**Table 1:** Current density of this work in comparison to previous works that harvest energy from water droplet.

Reference	Year	Current Density (*mA*/*m*^2^)
Moon *et al*.^21^	2013	6.4
Kwon *et al*.^22^ (drop)	2014	328.9
Kwon *et al*.^22^ (pushing/releasing)	2014	81.3
Lin *et al*.^8^	2015	26.3
Lin *et al*.^34^	2014	15.0
Cheng *et al*.^23^	2015	31.8
This work	2015	250

**Table 2:** Figure of merit of this work compared to some previous works on electrostatic generator devices.

Reference	Year	f(Hz)	S(*mm*^2^)	*V*_*C*_(*V*)	*P*(*μW*)	FOM ((10^8^*μW*)/(*mm*^2^*HzV*^2^))
Despesse *et al*.^10^	2005	50	1800	120	1050	81.02
Yen *et al*.^19^	2006	1560	4356	6	9.74	3.98
Tsutsumino *et al*.^14^	2006	20	200	950	37.7	1.04
Ma *et al*.^35^	2007	4100	25.9	15	0.065	0.27
Suzuki *et al*.^36^	2008	37	234	450	0.28	0.02
Basset *et al*.^17^	2008	250	66	8	0.061	5.78
Hoffmann *et al*.^18^	2008	1460	30	50	3.8	3.47
This work (500 mMNaCl)	2015	6	200	1.2	0.192	11111.11
This work (milliQ water)	2015	6	200	1.2	0.066	3819.44
